# Glycoprotein Biosynthesis in a Eukaryote Lacking the Membrane Protein Rft1*

**DOI:** 10.1074/jbc.M113.479642

**Published:** 2013-05-28

**Authors:** Jennifer Jelk, Ningguo Gao, Mauro Serricchio, Aita Signorell, Remo S. Schmidt, James D. Bangs, Alvaro Acosta-Serrano, Mark A. Lehrman, Peter Bütikofer, Anant K. Menon

**Affiliations:** From the ¶ Department of Biochemistry, Weill Cornell Medical College, New York, New York 10065,; the ‡ Institute of Biochemistry and Molecular Medicine, University of Bern, 3012 Bern, Switzerland,; the § Department of Pharmacology, University of Texas Southwestern Medical Center at Dallas, Dallas, Texas 75390,; the ‖ Department of Microbiology and Immunology, School of Medicine and Biomedical Sciences, State University of New York at Buffalo, Buffalo, New York 14214, and; the ** Parasitology and Vector Biology Departments, Liverpool School of Tropical Medicine, Liverpool L3 5QA, United Kingdom

**Keywords:** Endoplasmic Reticulum (ER), Glycobiology, Glycoprotein Biosynthesis, Lipid Synthesis, Membrane Proteins, Trypanosoma brucei, Flippase

## Abstract

Mature dolichol-linked oligosaccharides (mDLOs) needed for eukaryotic protein *N*-glycosylation are synthesized by a multistep pathway in which the biosynthetic lipid intermediate Man_5_GlcNAc_2_-PP-dolichol (M5-DLO) flips from the cytoplasmic to the luminal face of the endoplasmic reticulum. The endoplasmic reticulum membrane protein Rft1 is intimately involved in mDLO biosynthesis. Yeast genetic analyses implicated Rft1 as the M5-DLO flippase, but because biochemical tests challenged this assignment, the function of Rft1 remains obscure. To understand the role of Rft1, we sought to analyze mDLO biosynthesis *in vivo* in the complete absence of the protein. Rft1 is essential for yeast viability, and no Rft1-null organisms are currently available. Here, we exploited *Trypanosoma brucei* (Tb), an early diverging eukaryote whose Rft1 homologue functions in yeast. We report that TbRft1-null procyclic trypanosomes grow nearly normally. They have normal steady-state levels of mDLO and significant *N*-glycosylation, indicating robust M5-DLO flippase activity. Remarkably, the mutant cells have 30–100-fold greater steady-state levels of M5-DLO than wild-type cells. All *N*-glycans in the TbRft1-null cells originate from mDLO indicating that the M5-DLO excess is not available for glycosylation. These results suggest that rather than facilitating M5-DLO flipping, Rft1 facilitates conversion of M5-DLO to mDLO by another mechanism, possibly by acting as an M5-DLO chaperone.

## Teaser

**Background:** The membrane protein Rft1 was proposed to flip Man_5_GlcNAc_2_-PP-dolichol (M5-DLO) to the ER lumen for *N*-glycoprotein biosynthesis.

**Results:** Rft1-null *Trypanosoma brucei* has a normal steady-state level of mature dolichol-linked oligosaccharide (mDLO) and significant *N*-glycosylation.

**Conclusion:** Rft1 is not required for M5-DLO flipping *in vivo* but aids conversion of M5-DLO to mDLO by another mechanism.

**Significance:** The M5-DLO flippase remains to be identified.

## Introduction

Protein *N*-glycosylation is ubiquitous in eukaryotes. *N*-Linked oligosaccharides direct folding, quality control, and degradation of most proteins that enter the secretory pathway and also influence their subsequent trafficking (1). The importance of *N*-linked oligosaccharides is evinced by the discovery of human congenital disorders of glycosylation (2), in which errors in oligosaccharide synthesis or assembly result in developmental, neurological, and metabolic dysfunction, often with life-threatening consequences. Consequently, detailed knowledge of the mechanisms of *N*-linked oligosaccharide assembly is critically important for understanding human physiology.

**FIGURE 1.**
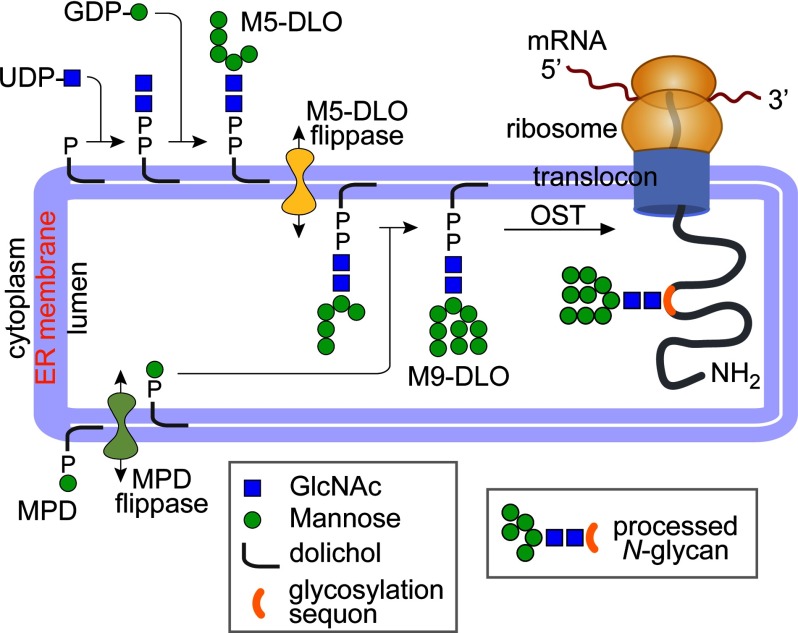
**DLO biosynthesis and protein *N*-glycosylation in *T. brucei* procyclic cells.** Dolichyl phosphate (*top left*) is sequentially glycosylated by UDP-GlcNAc- and GDP-Man-utilizing glycosyltransferases to generate M5-DLO on the cytoplasmic side of the ER. M5-DLO is translocated across the ER membrane by the M5-DLO flippase. In the ER lumen, it is extended to mature M9-DLO by mannosyltransferases that use mannose phosphate dolichol (*MPD*) as the mannosyl donor. Mannose phosphate dolichol (*bottom left*) is synthesized on the cytoplasmic face of the ER and flipped in by an mannose phosphate dolichol flippase to participate in luminal mannosyl transfer reactions. In procyclic form trypanosomes, oligosaccharyltransferase (*OST*) transfers an M9 glycan from mDLO to Asn residues of Asn-Xaa-(Ser/Thr) glycosylation sequons within ER-translocated proteins. The dolichyl diphosphate generated as a result is converted to dolichyl phosphate that then flips back across the ER to reinitiate DLO biosynthesis. *N*-Glycans are trimmed by luminal mannosidases to generate processed structures such as the example on the *right*.

The *N*-linked oligosaccharide moiety is assembled on a dolichyl diphosphate lipid carrier before being transferred *en bloc* to glycosylation sequons (Asn-Xaa-(Ser/Thr)) in nascent proteins as they enter the lumen of the endoplasmic reticulum (ER)^5^ (3–5). Synthesis of the dolichol-linked oligosaccharide (DLO) is a multistep process that initially generates Man_5_GlcNAc_2_-PP-dolichol (M5-DLO) on the cytoplasmic side of the ER (Fig. 1). M5-DLO is then flipped across the membrane to the ER lumen where it is elaborated in a series of reactions to mature DLO (mDLO). Metazoan mDLOs have a Glc_3_Man_9_GlcNAc_2_ glycan, although mDLOs in other eukaryotes have smaller glycans. For example, mDLO in procyclic forms of the parasitic protozoan *Trypanosoma brucei* has a Man_9_GlcNAc_2_ glycan (6) that, after transfer to protein, is trimmed by mannosidases to generate “processed” *N*-glycans (Fig. 1).

The molecular machinery required for DLO assembly has been largely identified, except for the lipid flippases responsible for translocating M5-DLO and other dolichol-based lipids across the ER membrane (Fig. 1) (3, 5, 7). This impasse appeared to be broken when Ng *et al.* (8) and later Helenius *et al.* (9). working with the yeast *Saccharomyces cerevisiae*, concluded that the ER membrane protein Rft1 is required directly for membrane translocation of M5-DLO. These investigators showed that yeast cells in which Rft1 expression was reduced to levels much lower than normal (though not nil) accumulate M5-DLO and hypoglycosylate proteins as would be expected for cells deficient in M5-DLO flippase activity. They also showed that overexpression of Rft1 alleviated the poor growth of *alg11*Δ cells that synthesize Man_3_GlcNAc_2_-PP-dolichol (M3-DLO) rather than M5-DLO on the cytoplasmic face of the ER. Within the framework of their proposal that Rft1 is a DLO flippase, they suggested that overexpression of Rft1 increased the predicted low rate of M3-DLO flipping to a level that could sustain the growth of *alg11*Δ cells.

The assignment of Rft1 as the M5-DLO flippase was challenged by biochemical studies. Sealed microsomes prepared from Rft1-depleted and Rft1-replete yeast cells were indistinguishable in their ability to synthesize mDLO, indicating that although Rft1 may have a critical role in mDLO biosynthesis *in vivo*, it was dispensable *in vitro* (10). Further concerns with the proposed role of Rft1 as the M5-DLO flippase were revealed by biochemical reconstitution experiments. M5-DLO flipping was demonstrated in proteoliposomes reconstituted with rat liver or yeast ER membrane proteins (11–13). Flipping in this reconstituted system was highly specific (13); a nonbiological structural isomer of M5-DLO was not flipped, and M6-DLO and larger DLO structures were flipped slowly, consistent with the early work of Snider and Rogers (14). Importantly, proteoliposomes lacking Rft1 were identical in their activity to Rft1-containing preparations, and biochemical fractionation of ER membrane protein extracts prior to reconstitution revealed that M5-DLO flippase activity could be resolved from Rft1 by a variety of methods, including velocity sedimentation and ion exchange chromatography (11, 12).

The cumulative data indicate that Rft1 is intimately involved in mDLO biosynthesis; however, its specific role in the pathway and its possible contribution to M5-DLO flipping remain enigmatic. To address this issue, we sought to analyze mDLO biosynthesis *in vivo* in the complete absence of the protein. As this could not be done in yeast where Rft1 is essential for viability under standard growth conditions, we turned to *T. brucei*, an early diverging eukaryote with an *N*-glycosylation pathway similar to that found in higher eukaryotes (15). *T. brucei* is a parasitic protozoan that causes sleeping sickness in humans and nagana in animals throughout sub-Saharan Africa. Compared with *S. cerevisiae* cells that grow rapidly and thus need *N*-glycosylation for osmoprotective cell wall assembly, we reasoned that the relatively slow-growing trypanosomes might be able to tolerate the absence of Rft1, especially if the protein provided an accessory rather than a core function in DLO biosynthesis. We identified the *T. brucei* Rft1 homologue and demonstrated that it provides the essential function of Rft1 in yeast. We now report that the procyclic form of *T. brucei* tolerates homozygous null disruptions of the *TbRft1* gene. Remarkably, the TbRft1-null strains synthesize normal levels of mDLO and transfer mDLO-derived glycans to protein but nevertheless accumulate M5-DLO and underglycosylate proteins. Our results show that M5-DLO flipping occurs in the complete absence of Rft1 *in vivo* but that Rft1 nonetheless influences the conversion of M5-DLO to M9-DLO.

## EXPERIMENTAL PROCEDURES

### 

#### 

##### Materials

Unless otherwise stated, all reagents were of analytical grade and purchased from Sigma or Merck. Restriction enzymes were from Fermentas (St. Leon-Rot, Germany) and antibiotics from Sigma, Invivogen (Nunningen, Switzerland), or Invitrogen. EasyTag^®^ Expre^35^S^35^S protein labeling mix was from PerkinElmer Life Sciences. BioMax MS and MXB films were from GE Healthcare.

##### Trypanosome Cultures

*T. brucei* strain Lister 427 procyclic forms were cultured at 27 °C in SDM-79 containing 5% heat-inactivated fetal bovine serum. Rft1 knock-out clones were grown under the same condition, in the presence of 15 μg/ml G418 for the single allele knock-out and an additional 10 μg/ml blasticidin for the double allele knock-out clones.

##### Generation of TbRft1-null Cells

Constructs to replace the endogenous *TbRft1* genes were based on the pKO plasmid containing resistance cassettes consisting of the following elements (5′ to 3′): the *EP1-EP2* procyclin intergenic region, a G418 or blasticidin resistance gene, and the tubulin βα intergenic region (16). A 397-bp 5′ and a 427-bp 3′ recombination sequence flanked the resistance cassettes. Recombination sequences adjacent to the TbRft1 open reading frame were obtained by PCR amplification using primers 5′_forward gcccaagcttacatgtcgctttaagttccgc and 5′_reverse gcgaatcccacaccaaaggtacagctgctgc for the 5′-recombination sequence and 3′_forward cgctctagagtggtgaaggcgctggttc and 3′_reverse gcggagctcagcttggagtccatgagtgg for the 3′-recombination sequence (HindIII, EcoRI, XbaI, and SacI restriction sites are underlined). Prior to transfection into *T. brucei* 427 procyclic forms, plasmids were digested upstream and downstream of the recombination sequences using HindIII and SacI. Clones were obtained by limiting dilution under antibiotic selection using 15 μg/ml G418 and/or 10 μg/ml blasticidin. Clones were PCR-tested for correct integration using primer 5′ UTR_control ggaagcgcaatcattcagag, which binds 50 bp upstream of the 5′ recombination sequence, in combination with different reverse primers.

To introduce an ectopic copy of Rft1 into TbRft1-null cells, *TbRft1* was amplified with primers TbRft1_forward gcccaagcttatggacttcaaacgacagctg and TbRft1_reverse gccctcgagctactcgccgcttctttttg and cloned into a pLEW100-based expression vector (17) (HindIII and XhoI restriction sites are underlined). Clones were obtained by limiting dilution under antibiotic selection using 2 μg/ml puromycin.

##### Southern Blot Analysis

For Southern blot analysis, 1.2 μg of AgeI or SacII/SmaI-digested genomic DNA from wild-type and mutant cells was separated on a 1% agarose gel and transferred to Hybond-N+ nylon transfer membrane (GE Healthcare) using 10× SSC buffer (150 mm Na_3_-citrate, pH 7.0, containing 1.5 m NaCl). The membrane was probed with a 427-bp ^32^P-labeled PCR product of the TbRft1 3′ recombination sequence generated with the prime-a-gene labeling system (Promega, Dübendorf, Switzerland). The hybridized probe was detected by autoradiography using BioMax MS films in combination with intensifying screens.

##### ^35^S Labeling of Trypanosomes and Immunoprecipitation of p67

Wild-type and TbRft1-null cells were labeled with EasyTag^®^ Expre^35^S^35^S essentially as described before (18, 19). Briefly, trypanosomes were washed twice in PBS and resuspended at 10^7^/ml in pre-warmed (27 °C) Met/Cys-deficient SDM-79 containing 5% dialyzed FBS for 10 min. Labeling was initiated by the addition of [^35^S]Met/Cys to 100 μCi/ml. After 60 min of incubation, cells were washed with cold buffer and resuspended in cold solubilization buffer (150 mm NaCl, 50 mm Tris-HCl, pH 8.0, containing 1% Nonidet-40, 0.1% SDS, and 0.5% deoxycholate). After centrifugation for 5 min at 1500 × *g* in a microcentrifuge, mouse anti-p67 was added to the clear supernatant and incubated under constant rotation for 60 min at 4 °C. Antibody-bound p67 was precipitated using protein A-agarose for 60 min at 4 °C under rotation, washed, and resuspended in electrophoresis buffer.

##### Enzyme Treatments

Parasite lysates or immunoprecipitated proteins were treated with *N*-glycosidase F (PNGase) or endoglycosidase H (Endo H) according to the manufacturer's instructions.

##### SDS-PAGE, Autoradiography/Fluorography

Parasite lysates or immunoprecipitated proteins were separated on 10% polyacrylamide gels under reducing conditions (20). ^35^S-Labeled proteins were detected after fixation of the gel and exposure to BioMax MS films at −70 °C.

##### Fluorophore-assisted Carbohydrate Electrophoresis (FACE) Analysis of Trypanosomal Glycoconjugates

Trypanosomes were collected by centrifugation and washed twice with ice-cold PBS. Methanol (room temperature) was added to the tube, and the contents were disrupted by vigorously vortexing. The suspensions were dried and processed for FACE analyses as described (21, 22). In brief, after most lipids were removed by extraction with chloroform/methanol (2:1, by volume) and aqueous components removed by extraction with water, DLOs were obtained by extraction with chloroform/methanol/water (10:10:3, by volume). *N*-Linked glycoproteins remained in the residual material. The glycan units of DLOs were released with weak acid. *N*-Glycans were released with PNGase F and further purified by ion exchange. All glycans were conjugated with 7-amino-1,3-naphthalenedisulfonic acid (ANDS) and resolved on an oligosaccharide profiling gel, with all loads normalized to total protein in the chloroform/methanol/water (10:10:3) residue. When necessary, the fluorescently conjugated glycans were incubated in 50 mm sodium citrate buffer, pH 5.5, for 18 h at 37 °C with 100 units of Endo H (New England Biolabs) and/or 0.05 milliunits of α-1,2-mannose-specific mannosidase (*Aspergillus saitoi*, Prozyme). Fluorescently labeled oligosaccharides were detected with a Bio-Rad Fluor-S scanner and quantified with Quantity-One software.

##### Flow Cytometry Analysis

Trypanosomes at mid-log phase (10^7^ parasites) were harvested by centrifugation at 1500 × *g* for 10 min at 4 °C and resuspended in 200 μl of cold SDM-79. Concanavalin A-FITC conjugate was added to a final concentration of 3 μg/ml. After 1 h of incubation in the dark on ice, 1 ml of cold SDM-79 was added, and parasites were centrifuged as above. After washing once in cold SDM-79, trypanosomes were resuspended in 2 ml of cold SDM-79 and analyzed by flow cytometry (BD FACSCalibur or FACScan) at a concentration of 5 × 10^5^ cells/ml. Data were analyzed using flow cytometry analysis software FlowJo.

##### Immunofluorescence Microscopy

Parasites were processed for immunofluorescence microscopy exactly as described before (23), using antibodies against p67 (19) and TbCatL (24) at dilutions of 1:1000 and 1:500, respectively. Secondary antibodies Alexa Fluor 594 goat α-mouse IgG (Invitrogen) and Alexa Fluor 488 goat α-rabbit IgG (Invitrogen) were used at dilutions of 1:1000.

## RESULTS

### 

#### 

##### T. brucei Rft1 Is Functionally Equivalent to S. cerevisiae Rft1

**FIGURE 2.**
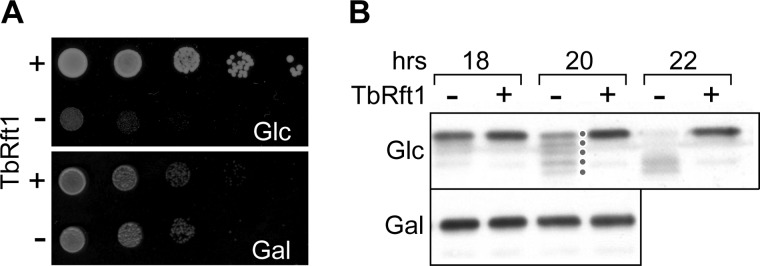
**TbRft1 is functionally equivalent to *S. cerevisiae* Rft1.** *A*, YG1137 yeast cells (in which Rft1 expression is controlled by the glucose-repressible *GAL1–10* promoter) were transfected with an episomal *URA3* plasmid encoding TbRft1 (+) or an empty vector (−). Serial dilutions were spotted on minimal uracil-free solid media containing either 2% glucose (*Glc*) or galactose (*Gal*), and incubated for 2 days at 30 °C. *B*, YG1137 cells were transfected as in *A* and grown on Glc- or Gal-containing minimal media at 30 °C. Extracts were prepared at the indicated times and analyzed by SDS-PAGE and immunoblotting with anti-carboxypeptidase Y (*CPY*) antibodies. Mature and hypoglycosylated forms of CPY, particularly evident in the 20 h sample from Glc-grown cells transfected with the empty vector, are indicated by *dots*.

We identified an Rft1 orthologue in *T. brucei* by BLAST searching the predicted proteome of *T. brucei* strain TREU 927 using human and *S. cerevisiae* Rft1 sequences as queries. We retrieved a single orthologous sequence, Tb11.01.3540, that was annotated as a 598-amino acid hypothetical protein. Topology prediction programs indicated that Tb11.01.3540 is a membrane protein with multiple transmembrane spans. To determine whether Tb11.01.3540 could functionally substitute for yeast Rft1 (ScRft1), we used a haploid yeast strain (YG1137) (9) in which ScRft1 is expressed under the control of the glucose-repressible *GAL1–10* promoter. On shifting YG1137 cells from galactose- to glucose-containing medium, ScRft1 expression is repressed and the cells fail to grow (Fig. 2*A*). At the same time, carboxypeptidase Y (CPY), a vacuolar protease with four *N*-glycans, becomes hypoglycosylated, with ever fewer glycosylation sites on the protein being occupied as the incubation time in glucose-containing media is increased and ScRft1 levels decrease (Fig. 2*B*, *lanes* indicated *TbRft1*−) (9–11). Heterologous expression of Tb11.01.3540 enabled YG1137 cells to grow on glucose-containing media (Fig. 2*A*) and prevented CPY hypoglycosylation (Fig. 2*B*, *lanes* indicated *TbRft1*+) indicating that the trypanosome protein, henceforth TbRft1, is functionally equivalent to ScRft1.

##### TbRft1 Is Nonessential in Procyclic Form Trypanosomes

**FIGURE 3.**
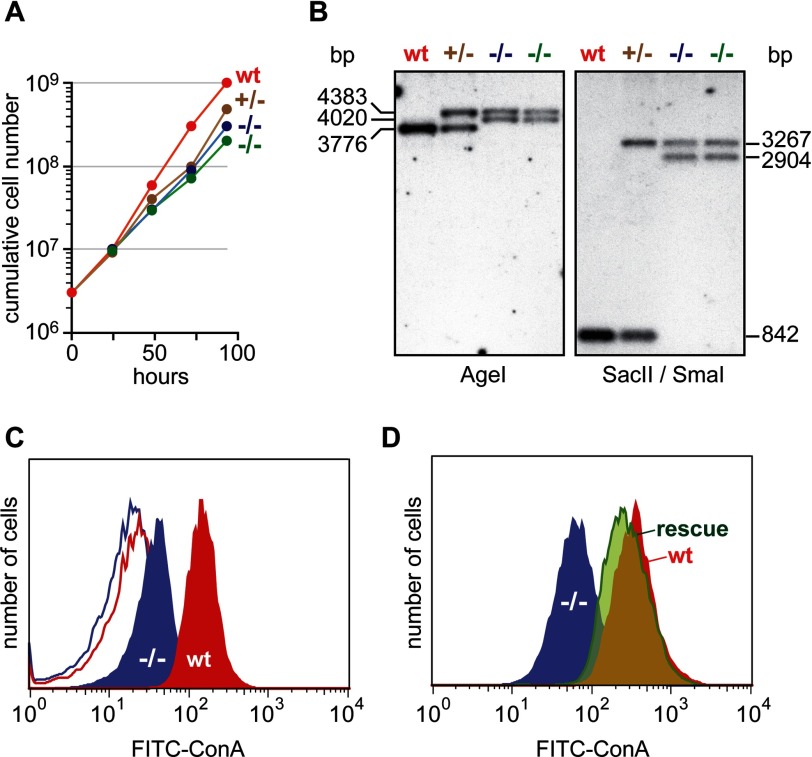
**Characterization of TbRft1-null trypanosomes.** *A*, cumulative growth of wild-type and mutant *T. brucei* procyclic forms. *B*, Southern blot analysis. AgeI or SacII/SmaI-digested genomic DNA from wild type and mutant trypanosomes was separated on an agarose gel, transferred to hybond-N+ nylon transfer membrane, and probed with a 427-bp ^32^P-labeled PCR product of the TbRft1 3′ recombination sequence. The hybridized probe was detected by autoradiography. *C*, ConA reactivity. Wild-type (*wt*) and TbRft1-null (−/−) trypanosomes were fixed and incubated with or without FITC-ConA. The extent of labeling was determined by flow cytometry. The corresponding profiles of unlabeled cells are shown as *line traces*, without *fill. D*, flow cytometry analysis of TbRft1-null cells expressing TbRft1 (*rescue*) compared with TbRft1-null and wild-type trypanosomes. Analysis was done as in *C*.

We used homologous recombination to replace the *TbRft1* alleles in diploid procyclic cells with G418 and blasticidin resistance genes, eliminating the ability to express any form of TbRft1 protein. We recovered viable clones of TbRft1-null trypanosomes that grew nearly normally (Fig. 3*A*). The generation time of two independent TbRft1-null clones was 15.3 ± 0.9 and 14.4 ± 0.2 h, compared with 11 ± 0.2 and 13.1 ± 0.5 h for wild-type cells and single knock-out cells, respectively. These data suggest that growth rate is sensitive to *TbRft1* gene dosage (in contrast to effects on glycosylation, see below). Southern blot analyses (Fig. 3*B*) confirmed that we had indeed disrupted both alleles of *TbRft1* and that the growth of the cells was not due to inappropriate integration of the drug resistance genes elsewhere in the genome rather than at the *TbRft1* locus. We conclude that TbRft1 is a nonessential protein in *T. brucei* procyclic forms in culture.

To determine whether the TbRft1-null trypanosomes had a glycosylation defect, we incubated the cells with FITC-ConA, a fluorescent conjugate of the mannose-binding lectin concanavalin A, and quantified cell surface fluorescence by flow cytometry. TbRft1-null trypanosomes bound ∼75% less FITC-ConA than wild-type cells (Fig. 3*C*). This was specifically due to the lack of Rft1, as FITC-ConA binding could be restored by ectopic expression of TbRft1 (Fig. 3*D*, *histogram* labeled *rescue*). Thus, despite their robust growth characteristics, the Rft1-null cells show decreased levels of cell surface glycosylation.

##### Dolichol-linked Oligosaccharide Synthesis and Protein N-Glycosylation in TbRft1-null Cells

To examine the glycosylation phenotype of the Rft1-null trypanosomes in more detail, we used FACE (21), a sensitive method to quantify steady-state levels of oligosaccharides. The cells were treated with organic solvent to extract DLOs and precipitate proteins. Oligosaccharides were released from DLOs and *N*-glycoproteins with mild acid and peptide:*N*-glycosidase F (PNGase F) treatment, respectively. *N*-Glycans were further purified by ion exchange chromatography to enrich neutral glycans, including high mannose structures. All glycans were conjugated with ANDS fluorophore and analyzed on an oligosaccharide profiling gel.

**FIGURE 4.**
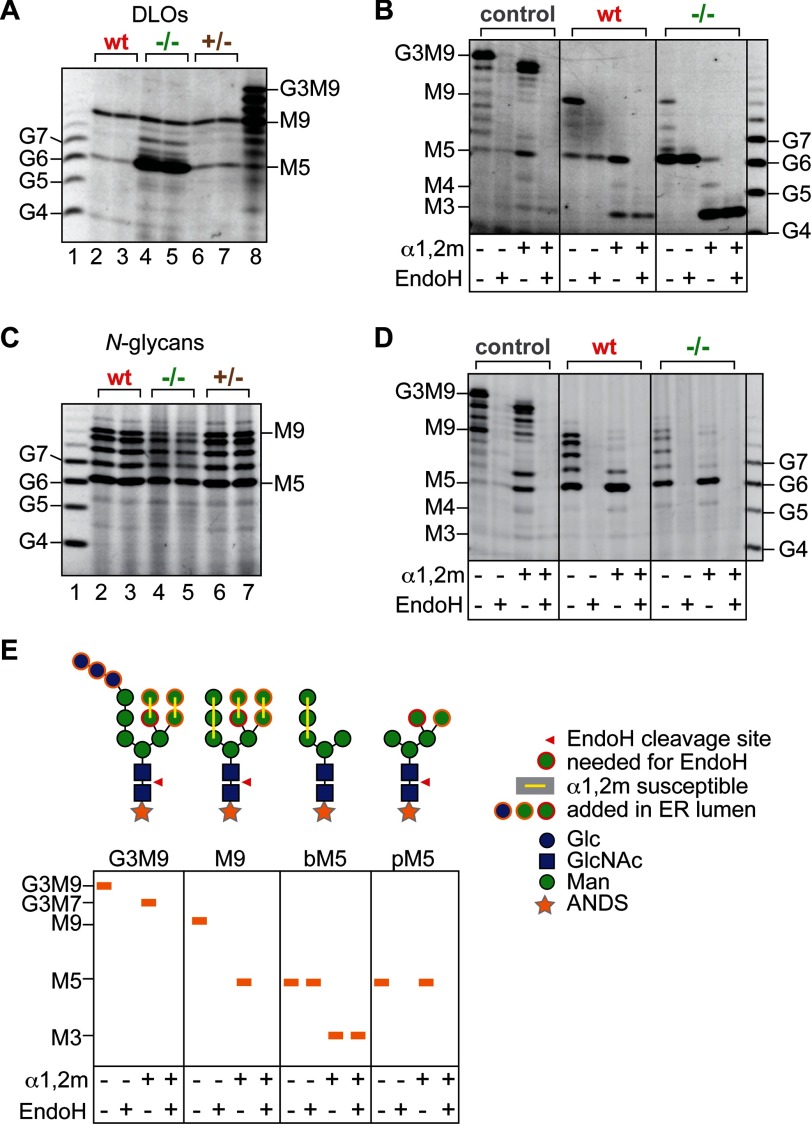
**M9-DLO is synthesized and used for *N*-glycosylation in TbRft1-null cells while biosynthetic M5-DLO accumulates.** DLOs and total neutral *N*-glycans from wild-type (*wt*), TbRft1-heterozygous (+/−), and TbRft1-null (−/−) trypanosomes were analyzed by FACE. The positions of glucose oligomers (*G4* to *G7*) and dolichol-linked glycan standards (*M3-G3M9*) are shown. *A*, DLOs from duplicate samples of wild-type (*lanes 2* and *3*), TbRft1-null (*lanes 4* and *5*), and TbRft1-heterozygous (*lanes 6* and *7*) cells. Glycans were released from the DLO fraction and labeled with ANDS prior to analysis. *B*, DLOs from rodent liver (*control*), or wild type (*wt*), or TbRft1-null trypanosomes. ANDS-labeled glycans were incubated in the absence or presence of Endo H or α1,2m as indicated. Cleavage by Endo H releases GlcNAc-ANDS, with loss of fluorescent label from the parent glycan. *C*, total neutral *N*-glycans, as for *A. D*, analysis of total neutral *N*-glycans by glycosidase digestion, as for *B*, except that the control used was a DLO preparation from rodent kidney, which has a relatively high proportion of nonglucosylated DLOs. *E*, schematic showing the glycosidase digestion pattern obtained for various glycans. *bM5*, biosynthetic biantennary Man_5_GlcNAc_2_; *pM5*, processed triantennary Man_5_GlcNAc_2_ (see also Fig. 1).

The profile of DLOs (Fig. 4*A*) in TbRft1-null trypanosomes was striking. The steady-state level of M9-DLO in the TbRft1-null cells (24.2 ± 2.2 pmol/mg) was similar to that in wild-type cells (22.8 ± 0.2 pmol/mg) indicating that the null cells have an intact DLO assembly pathway. However, the steady-state level of M5-DLO in the TbRft1-null trypanosomes was >40-fold higher than that in wild-type cells (382.7 ± 34 *versus* 8.7 ± 0.9 pmol/mg). Also apparent was the accumulation of M6-DLO, M7-DLO, and M8-DLO (Fig. 4*A*), whose consumption has no obvious requirement for the M5-DLO flippase. Cumulative results obtained from independent experiments and individual TbRft1-null clones (Table 1) indicate that this pattern is highly reproducible; TbRft1-null cells have a 30–100-fold higher steady-state level of M5-DLO compared with wild-type cells but an essentially normal level of M9-DLO (Table 1). Although a reduction in *TbRft1* gene dosage had a mild effect on the rate of growth (Fig. 3*A*), steady-state levels of the various glycoconjugates were similar in wild-type and heterozygous trypanosomes (Table 1).

**TABLE 1:** **Steady-state levels of M9-DLO, M5-DLO, total DLOs and total neutral *N*-glycans in wild-type, TbRft1-null, and single knock-out trypanosomes** Mean ± range of duplicate measurements is quoted as picomoles of glycoconjugate per mg of protein; ND means not determined. Two different TbRft1-null clones (A3 and B1(2)) were analyzed, alongside single knock-out (3F) and wild-type (427) cells. Raw data for experiment 1 are depicted in Fig. 3, *A* and *C*.

Experiment	Glycoconjugate	427 (+/+)	A3 (−/−)	B1(2) (−/−)	3F (+/−)
1	M9-DLO	22.8 ± 0.2	24.2 ± 2.2	ND	24.2 ± 1.6
	M5-DLO	8.7 ± 0.9	382.7 ± 34	ND	12.3 ± 0.5
	Σ DLO	32.2 ± 1.4	423.7 ± 36	ND	40.4 ± 2.2
	Σ *N*-glycans	687 ± 16	294 ± 36	ND	727 ± 2
2	M9-DLO	17.4 ± 0.6	6.4 ± 0.8	14.2 ± 1.8	25.2 ± 0.4
	M5-DLO	8.4 ± 0.4	248.2 ± 2.2	840.8 ± 0.4	28.6 ± 3.2
	Σ DLO	25.8 ± 1	254.6 ± 3	855 ± 2.2	54 ± 2.8
	Σ *N*-glycans	857 ± 100	323 ± 27	454 ± 16	907 ± 82

We considered whether the accumulated M5-DLO is a direct product of the DLO biosynthetic pathway (“biosynthetic M5” (Fig. 4*E*)) or whether it is derived from mature M9-DLO as a result of the action of cellular mannosidases (“processed M5” (Fig. 4*E*)). To distinguish these possibilities, we determined the susceptibility of the accumulated M5-DLO to Endo H and α1,2-mannosidase (α1,2m). As outlined schematically in Fig. 4*E*, biosynthetic M5 is susceptible to α1,2m yielding Man_3_GlcNAc_2_-ANDS but resistant to Endo H; in contrast, processed M5 is susceptible to Endo H (the GlcNAc-ANDS product is not retained within the resolving gel) but resistant to α1,2m. The glycosidase digests shown in Fig. 4*B* indicate clearly that the accumulated M5-DLO is “biosynthetic,” *i.e.* it corresponds to a DLO biosynthesis intermediate.

We next analyzed neutral *N*-glycans released by PNGase F. *T. brucei* procyclic forms almost exclusively make high mannose type *N*-glycans (25). The profile of *N*-glycans seen in TbRft1-null trypanosomes was identical to that in wild-type cells except that the total level of *N*-glycans was, on average, ∼67% lower (Fig. 4*C* and Table 1), consistent with the ∼75% lower surface reactivity of TbRft1-null cells toward ConA (Fig. 3*C*). The full range of *N*-glycans was observed in TbRft1-null cells, from Man_5_GlcNAc_2_ to Man_9_GlcNAc_2_. Glycosidase digests (Fig. 4*D*, illustrated schematically in Fig. 4*E*) indicated that the Man_5_GlcNAc_2_ species recovered in the *N*-glycan fraction corresponds to processed M5 (Fig. 4*E*). This result indicates that in Rft1-null cells a higher order oligosaccharide such as M9 is transferred to proteins and subsequently trimmed by cellular mannosidases to processed M5. The cumulative data reveal that TbRft1-null trypanosomes have a complete *N*-glycosylation pathway but nevertheless accumulate large amounts of the M5-DLO biosynthetic intermediate that is not directly transferable to protein.

##### N-Glycosylation of p67 in TbRft1-null Cells

**FIGURE 5.**
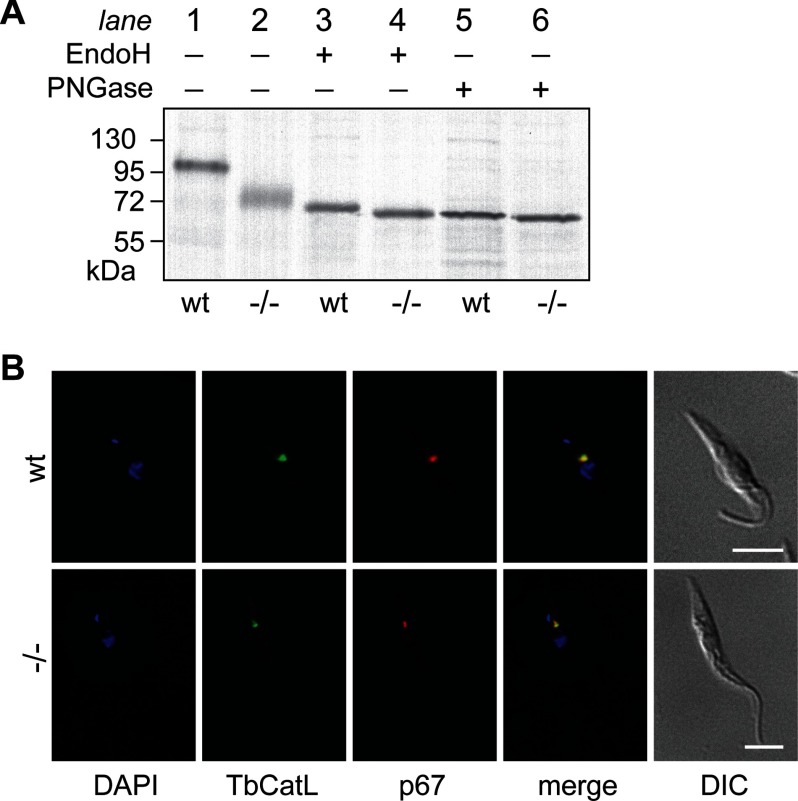
**Analysis of p67.** *A*, analysis of p67 from wild-type and TbRft1-null cells. Trypanosomes were metabolically labeled with ^35^S-labeled amino acids and lysed, and p67 was immunoprecipitated and analyzed by SDS-PAGE and fluorography, either directly or after Endo H or PNGase F treatment. *B*, fluorescence micrographs obtained using DAPI, as well as antibodies against p67 and TbCatL. DAPI staining reveals kinetoplast and nuclear DNA. *DIC*, differential interference contrast.

To refine our analyses of *N*-glycosylation in the TbRft1-null trypanosomes, we focused on a specific protein and chose p67, a well characterized lysosomal *N*-glycoprotein with 14 *N*-glycosylation sites (19). We immunoprecipitated p67 from trypanosomes that had been metabolically labeled with [^35^S]methionine/cysteine and analyzed the protein by SDS-PAGE and fluorography. Fig. 5*A* shows the expected ∼100-kDa band for fully glycosylated p67 in wild-type cells and a lower molecular weight (∼72 kDa) diffuse band in TbRft1-null cells (Fig. 5*A*, compare *lane 2* with *lane 1*). Treatment with either Endo H or PNGase F converted the diffuse band to an ∼67-kDa product (Fig. 5*A*). Thus, p67 in TbRft1-null cells is *N*-glycosylated, and the glycans are fully Endo H-sensitive indicating that they have the expected oligomannose structure.

We observed that Endo H-treated p67 from TbRft1-null trypanosomes migrates slightly faster than the corresponding sample from wild-type cells (Fig. 5*A*, compare *lane 4* with *lane 3*). This is likely because fewer *N*-glycosylation sites are occupied in p67 from TbRft1-null cells, and therefore, there are fewer GlcNAc residues left on the protein after Endo H cleavage. Differences in migration are also observed in PNGase F-treated p67 samples (Fig. 5*A*, compare *lane 6* with *lane 5*). In this case, lower site occupancy would result in fewer Asn residues being converted into negatively charged Asp residues after PNGase F digestion of p67 from TbRft1-null trypanosomes, making this protein migrate slightly faster than its counterpart from wild-type cells. We conclude that fewer *N*-glycosylation sites are occupied in p67 from TbRft1-null cells compared with wild-type cells, thus explaining its faster and more diffuse migration on SDS-PAGE. Based on gel mobility, we estimate that p67 in the TbRft1-null trypanosomes has 2–4 *N*-glycans.

Despite being underglycosylated, p67 is correctly localized to the lysosome in TbRft1-null cells. Immunofluorescence microscopy (Fig. 5*B*) revealed a single organelle in the region between the nucleus and the posterior flagellar pocket that was stained with anti-p67 antibodies as well as with antibodies against TbCatL, a trypanosomal cathepsin L orthologue (24).

## DISCUSSION

We generated an Rft1-null cell, making it possible for the first time to characterize quantitatively the consequences of complete Rft1 absence by measuring the steady-state levels of key DLO intermediates and *N*-glycoproteins. In contrast, previous analyses of Rft1 function *in vivo* were done on cells in which the protein was acutely depleted but not eliminated (9). Our results clearly show that TbRft1-null procyclic trypanosomes can execute the entire ER *N*-glycosylation pathway, *i.e.* synthesis of M9-DLO, transfer of Man_9_GlcNAc_2_ to proteins, and trimming of *N*-glycans. However, measurements of the steady-state levels of DLO intermediates by FACE show that although M9-DLO levels are unaffected, the mutant cells accumulate large amounts of the M5-DLO biosynthetic intermediate. Accumulation of the downstream intermediates M6-DLO, M7-DLO, and M8-DLO was also detected. These data indicate that TbRft1 impacts the multistep conversion of M5-DLO to M9-DLO.

Our results are inherently inconsistent with the assignment of Rft1 (8, 9) as the M5-DLO flippase. Another protein, yet to be identified, very likely plays this role because spontaneous flip-flop of M5-DLO (26) is too slow to supply the DLO biosynthetic pathway in TbRft1-null trypanosomes; the *t*½ for M5-DLO flipping in synthetic bilayers is predicted to be ≫100 h, *i.e.* much slower than that for common phospholipids (27). It is formally possible that M5-DLO flipping in the mutant cells is the result of a nonspecific process, involving membrane factors that do not normally serve this function. However, given our earlier demonstration of a chromatographically discrete protein fraction with structure-specific M5-DLO flipping activity *in vitro* (11–13), there is little justification for invoking such a model.

For insights into the observed steady-state levels of M5-DLO and M9-DLO in wild-type and TbRft1-null cells, we modeled the DLO pathway as a sequential, irreversible series of reactions in which a source (*S*) is converted to M5-DLO, then to M9-DLO, and finally to product(s) (*P*). This simplification is possible because in steady state, a series of reactions can be replaced by a single reaction without invoking a rate-limiting step. Thus, in Reaction 1, 
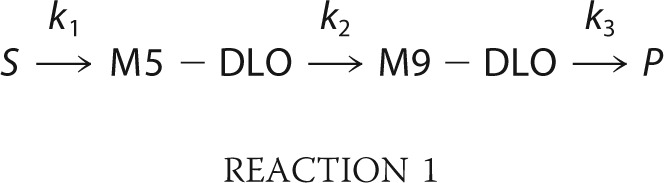
 where the rate constant *k*_1_ describes the set of reactions leading to the production of M5-DLO, the rate constant *k*_2_ characterizes the multistep conversion of M5-DLO to M9-DLO, and the rate constant *k*_3_ indicates the conversion of M9-DLO to products. The steady-state levels of M5-DLO and M9-DLO are shown in Equation 1, 
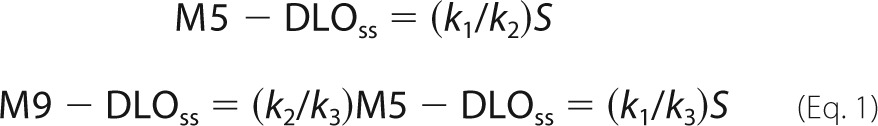
 Thus, the simplest explanation for the ∼100-fold higher level of M5-DLO_ss_ in TbRft1-null cells is that *k*_2_ is ∼100-fold lower in these cells compared with wild-type cells. This change does not affect M9-DLO_ss_, which does not depend on *k*_2_.

How does TbRft1 affect *k*_2_, *i.e.* conversion of M5-DLO to M9-DLO? The processes encapsulated in *k*_2_ are minimally the M5-DLO flipping event and the four luminal mannosyl transfer reactions. As the cumulative biochemical data reported earlier (10–12) and the *in vivo* results presented here demonstrate that M5-DLO flipping does not require Rft1, and the luminal mannosyltransferases are known (Alg3, Alg9, and Alg12, with Alg9 carrying out two of the four mannosylations (7)), we conclude that Rft1 carries out its role separately from the core machinery required for conversion of M5-DLO to M9-DLO. We suggest that Rft1 plays an accessory albeit important role in M9-DLO biosynthesis. For example, it may control *k*_2_ by functioning as a DLO chaperone to supply M5-DLO to the flippase or mannosyltransferases or to increase the chemical activity of M5-DLO by preventing its possible aggregation. Similar considerations apply to M6-, M7-, and M8-DLO as these lipids also accumulate in the TbRft1-null cells. Alternatively, it could stabilize a complex of the luminal mannosyltransferases, thus enabling substrate channeling. Complex formation involving components of the DLO biosynthetic machinery has been previously reported (28, 29). In this context, it is interesting to note that disease-causing mutations in human Rft1 are located in hydrophilic loop regions of the protein that are predicted to be in the ER lumen (30); these functionally important loops could conceivably be involved in Rft1's proposed role as luminal M5-DLO chaperone or its proposed role as a partner of luminal mannosyltransferases. As discussed previously (11, 12), Rft1's function may resemble that of Lec35/MPDU1, a metazoan protein that is involved in luminal mannosyl transfer reactions *in vivo* (31). Lec35/MPDU1 was originally thought to be an excellent candidate for the mannose-phosphate dolichol flippase (Fig. 1), but the current consensus is that it acts as a dolichyl-lipid chaperone (31, 32).

Why is *N*-glycosylation reduced even though M9-DLO_ss_ levels are normal in TbRft1-null cells? Steady-state measurements do not give information about flux. Thus, although M9-DLO_ss_ levels are normal, the rates of M9-DLO synthesis and consumption may be coordinately reduced by factors (yet to be determined) secondary to the absence of TbRft1. M9-DLO is subject to at least two known fates (collectively described by the rate constant *k*_3_ in Reaction 1), consumption by oligosaccharyltransferase for protein *N*-glycosylation and turnover, possibly by regulated cleavage of its pyrophosphate linkage (22). The balance between the two fates can be altered without affecting *k*_3_ or M9-DLO_ss_. Thus, with less M9-DLO being consumed for *N*-glycosylation, greater turnover could be accommodated in the TbRft1-null strain. However, it is unlikely that mannose 6-phosphate-dependent hydrolysis of DLOs described by Gao *et al.* (22, 33) could contribute to DLO turnover because M9-DLO appears not to be a substrate for this process. Alternatively, M9-DLO levels could be limiting in both wild-type and TbRft1-null trypanosomes. In this event, the reduced rate of production of M9-DLO in the TbRft1-null cells could account for the observation of fewer glycans per protein.

Where does M5-DLO accumulate in TbRft1-null cells? Wild-type procyclic trypanosomes normally transfer M9 oligosaccharides to proteins and process these to triantennary M5 structures (Fig. 1). When biosynthetic M5-DLO is the only donor available to oligosaccharyltransferase in procyclic forms of *T. brucei*, proteins are underglycosylated and *N*-glycans are aberrantly processed to biantennary complex structures (34). The M5-DLO that accumulates in TbRft1-null cells could potentially compete with mature M9-DLO in the oligosaccharyltransferase reaction, resulting in biantennary complex *N*-glycans that would be resistant to Endo H digestion. This is not the case; analysis of total *N*-glycans (Fig. 4*D*), as well as *N*-glycans on p67 (Fig. 5*A*), indicates structures that are completely susceptible to Endo H. The M5 structures that we detected in *N*-glycans were also Endo H-sensitive; they were derived by normal processing of mature M9 *N*-glycans and did not originate by direct transfer of biosynthetic M5. Thus, M5-DLO does not compete with M9-DLO and therefore must not have access to the site of oligosaccharyl transfer.

Either of two contrasting models could account for the segregation of accumulated M5-DLO from the site of oligosaccharyl transfer in the ER: model 1, M5-DLO accumulates on the cytoplasmic side of the ER, and model 2, M5-DLO accumulates on the luminal side of the ER, perhaps in an aggregated form with low chemical activity or in a region of the ER that is laterally segregated from the oligosaccharyltransferase. Examples of lateral segregation of reactions within a single pathway in the ER have been previously noted (35, 36). The models have implications for possible functions of Rft1. In model 1, Rft1 would function as an accessory protein to supply M5-DLO to the flippase on the cytoplasmic side of the ER, and in model 2, Rft1's function would be on the luminal side of the ER, possibly to prevent M5-DLO aggregation and increase its chemical activity, or chaperone M5-DLO between ER domains.

The two models predict different transbilayer orientations for M5-DLO; in model 1, M5-DLO would be mainly oriented toward the cytoplasm, and in model 2, M5-DLO would be able to flip bidirectionally between leaflets. Current biochemical approaches, *e.g.* capturing M5-DLO on the cytoplasmic side of intact ER preparations with a lectin (11, 14), would not be able to distinguish between these models as M5-DLO would be quantitatively captured in both cases. Indeed, unless it is known that the transbilayer distribution of M5-DLO is stable during the analysis, it would be difficult to make any conclusions about its orientation. Development of suitable techniques to distinguish between these models remains an objective for future work.

## Footnotes

5 The abbreviations used are:

ER: endoplasmic reticulum
M5-DLO: Man_5_GlcNAc_2_-PP-dolichol
mDLO: mature dolichol-linked oligosaccharide
PNGase F: *N*-glycosidase F
FACE: fluorophore-assisted carbohydrate electrophoresis
Endo H: endoglycosidase H
α1,2m: α1,2-mannosidase
ANDS: 7-amino-1,3-naphthalenedisulfonic acid
CPY: carboxypeptidase Y
ConA: concanavalin A.
